# First imported *Plasmodium ovale* malaria in Central America: case report of a Guatemalan soldier and a call to improve its accurate diagnosis

**DOI:** 10.1186/s40779-015-0030-9

**Published:** 2015-02-15

**Authors:** María Eugenia Castellanos, Sheilee Díaz, Emily Parsons, Leonard F Peruski, Fabiola Enríquez, Juan Luis Ramírez, Norma Padilla

**Affiliations:** Center for Health Studies-Universidad del Valle de Guatemala (CES-UVG), 18 Av. 11-95 zona 15, Vista Hermosa III, Guatemala City, CA Guatemala; National Reference Laboratory, Ministry of Health and Welfare, Km. 22, Carretera al Pacífico, Barcenas, Villa Nueva, CA Guatemala; F. Edward Hebert School of Medicine, Uniformed Services, University of the Health Sciences, 4301 Jones Bridge Road, 20814 Bethesda, MD USA; Global Disease Detection Program, Centers for Disease Control and Prevention (CDC), Central America Regional Office, 1600 Clifton Road NE, MS-D68, 30333 Atlanta, GA USA; Military Healthcare and Social Services, Ministry of Defense, Guatemala. Finca El Palomar, Acatan, Sta. Rosita Zona 16, Guatemala City, Guatemala; Military Medical Center, Ministry of Defense, Guatemala. Finca El Palomar, Acatan, Sta. Rosita Zona 16, Guatemala City, Guatemala

**Keywords:** Plasmodium ovale, Imported malaria, Diagnosis

## Abstract

The Mesoamerican Ministers of Health have set 2020 as the target for malaria elimination to be achieved in the region. Imported malaria cases are a potential threat to countries attempting elimination or working to prevent resurgence. We report the first imported *Plasmodium ovale* infection with molecular confirmation in Central America, which occurred in a Guatemalan soldier that had been deployed in Africa. The obstacles for its diagnosis using the standard microscopy technique and the need to improve its detection are discussed.

## Background

One factor in the worldwide increase of imported malaria cases has been the presence of armed forces and United Nations (UN) peacekeeping missions in malaria-endemic areas and their subsequent return to their countries of origin [1]. As expected, the majority of the cases are caused by *Plasmodium falciparum* or *P. vivax*. However, *P.ovale* infection has been established as a relevant cause of imported malaria in these groups, resulting in challenges for accurate diagnosis and treatment [2].

*P. ovale* is mainly distributed in sub-Saharian Africa and the islands of western Pacific with prevalence between 3-10% depending on the region, population and type of diagnostic tests used [3,4]. Recently, Sutherland *et al*. demonstrated there are two non-recombining sympatric species of *P. ovale*, named P*. ovale curtisi* (classic type) and *P. ovale* (variant type) [5]. In Latin America, no autochthonous *P. ovale* cases have occurred and there have been just a few reports of imported *P. ovale* infections [6-8].

Guatemala is a Central American country, with approximately 80% of its territory considered a malaria transmission setting [9]. *P. vivax* and *P. falciparum* are the etiologic agents of malaria, with the last case of *P. malariae* reported in 1971 and no *P. ovale* cases ever documented in Guatemala [10]. In the last decade, and largely based on the scale up of vector-control strategies, a significant reduction in the number of malaria cases in the country had been observed, from 39,703 cases in 2005 [11] to 3,292 cases in 2013 (Guatemalan Malaria Control Program, unpublished data). The same trend has been observed in all Mesoamerican countries and the Española Islands (Dominican Republic and Haiti) (Malaria Control Programs, unpublished data). Thus, malaria programs in Guatemala and Mesoamerica are now aiming towards malaria elimination rather than only prevention and control. However, imported cases pose a threat to this goal [12].

Since 2004, Guatemala has deployed military personnel to contribute to UN peacekeeping missions. In 2010, an epidemiological investigation detected 12 cases of imported *P. falciparum* malaria in 150 Guatemalan soldiers returning from the Democratic Republic of the Congo (DRC) [13,14]. Since this event, molecular screenings to detect *P. vivax* /*P. falciparum* malaria carriers are conducted annually among returning contingents from the DRC as part of a program established between the Guatemalan National Reference Laboratory, Universidad del Valle de Guatemala and Military Healthcare and Social Services. Here we present the first *P. ovale* case detected in this population. To the best of our knowledge this is the first report of molecularly-confirmed imported *P. ovale* infection in Central America. The challenges that this case presented for an accurate diagnosis and the need to incorporate more sensitive methods will be discussed.

## Case presentation

A 38-year-old male Guatemalan soldier presented to the facilities of the Military Hospital on June 29, 2012, to confirm suspected *P. vivax* malaria. Three months previously he had returned from a 9-month deployment in Africa, as part of a Guatemalan Army operation to aid the UN Organization Stabilization Mission in DRC.

The soldier reported that during his time in DRC he stayed in the towns of Dungu, Bangadi and Bunia. He travelled for a short period of time to Entebbe and Kampala (Uganda) and Mombasa (Kenya) as part of his rest and recuperation time. During the entire mission, he used an untreated bed net and received mefloquine prophylaxis (250 mg base/week). He reported high compliance with the drug, although he admitted to skipping some doses during his period in Uganda and Kenya. Upon his return to Guatemala, he and all deployed personnel were screened by malaria microscopy and *P.vivax*/*P.falciparum* PCR. Microscopic and molecular results were negative for the entire contingent.

Two months after his return to Guatemala, the patient travelled to Puerto de San José, Escuintla (a coastal zone in the southern Guatemala) and then returned to his residence in Jalpatagua, Jutiapa (eastern region). Malaria is endemic in both of these regions. He reported fatigue and fever, and malaria was clinically diagnosed by a private physician without any laboratory confirmation. He was given chloroquine diphosphate injections (no available dosage data) because the standard treatment in *P. falciparum*/*P. vivax* (the two endemic malaria parasites of the country) includes chloroquine [15].

The patient reported an improvement and returned to military duties. Three weeks later, he again presented with fever and fatigue. Malaria microscopy conducted at the Jutiapa Malaria Vector Control Program erroneously diagnosed a *P. vivax* infection. The patient was transferred to Guatemala City to be evaluated in the Military Medical Center.

Venous and capillary blood was taken for laboratory testing. A thin blood film identified *P. vivax,* although concerns about the morphology of some of the observed parasites were raised by the hospital microbiologist. A complete blood count at admission revealed thrombocytopenia (71,000 platelets/μl) and anemia (haemoglobin 12 g/dl). A rapid dengue diagnostic assay showed a current or recent infection. Other laboratory tests results were within reference values.

**Figure 1:**
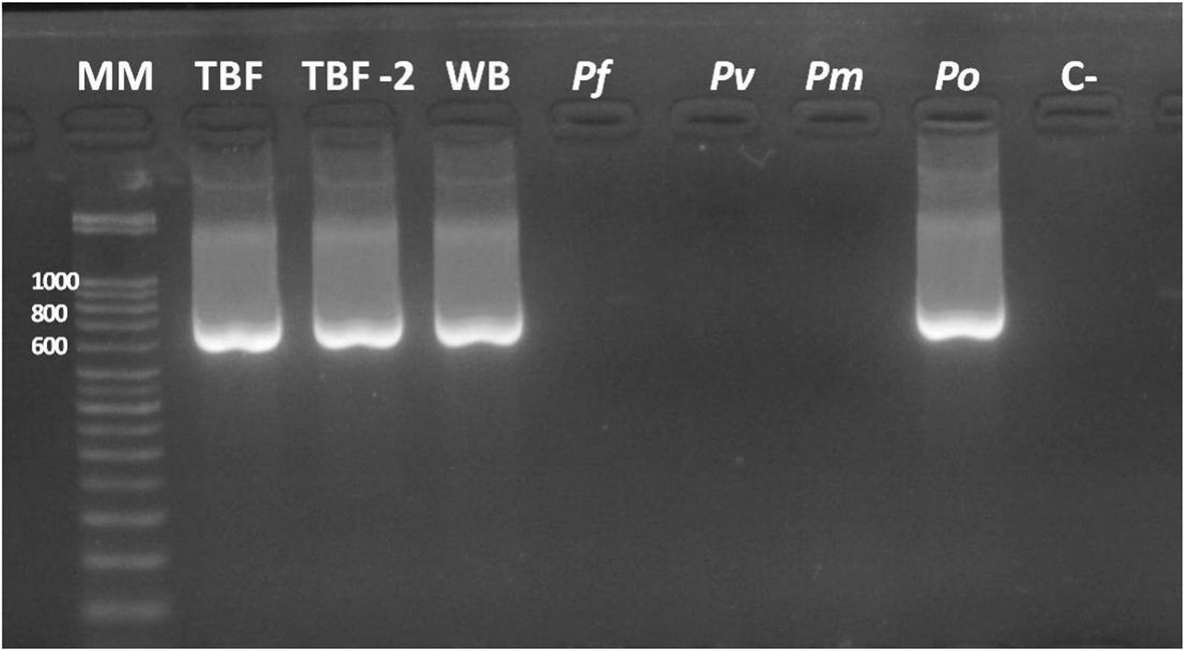
**Molecular detection of** ***Plasmodium ovale*** **in blood samples from patient by the amplification of 18S gene.** Nested PCR with *Plasmodium* genus-conserved primers was followed by *P. ovale* specific primers. PCR products were separated by electrophoresis through a 2.0% agarose gel stained with ethidium bromide and flanked by a 50 bp DNA ladder (Novagen) as a size marker. **Lane MM:** Molecular marker 50 bp. **Lane TBF:** Patient’s thin blood film. **LaneTBF-2**: Patient’s thin blood film (duplicate). **Lane WB:** Patient’s whole blood. **Lane** ***Pf*** **:** *Plasmodium falciparum* control. **Lane** ***Pv*** **:** *Plasmodium vivax* control. **Lane** ***Pm*** **:** *Plasmodium malariae* control. **Lane Po:** *Plasmodium ovale* positive control. **Lane C-:** Negative control.

**Figure 2:**
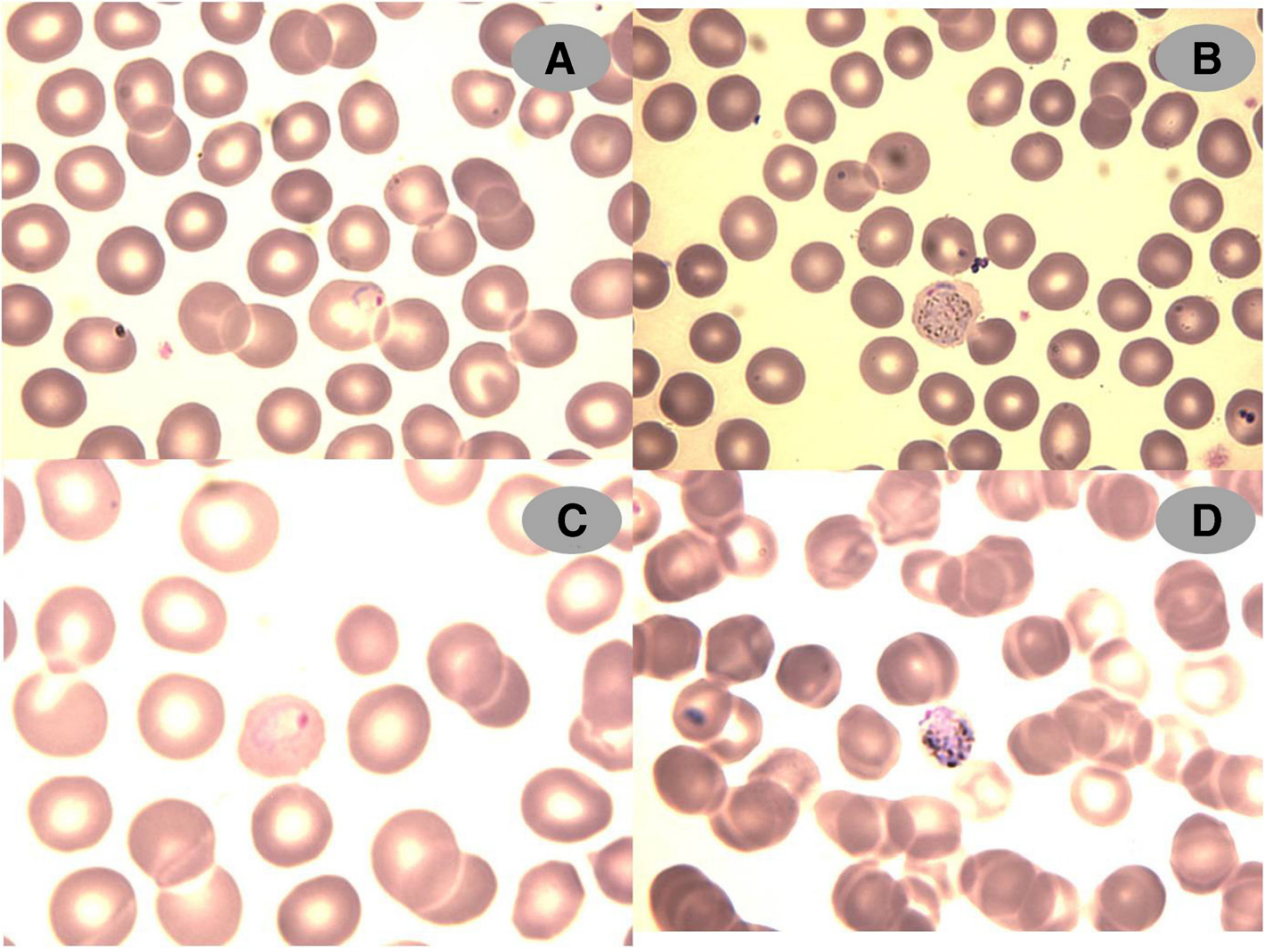
***Plasmodium ovale*** **parasites (initially diagnosed as** ***P. vivax*** **) shown in different stages in the blood smear of the patient at time of presentation (Giemsa Stain, X 1000). A** and **C**: ring-form trophozoites. **B** and **D**: gametocytes.

Based on the travel history of the patient and the doubts arisen as to its species identity, molecular testing was conducted to confirm the *Plasmodium* species. A *P. ovale* mono-infection was identified on both the blood film and whole blood of the patient using a *Plasmodium* genus-specific nested PCR (Figure 1) and a SYBR Green-based quantitative real-time PCR [16,17]. The blood film was re-examined to identify the morphological features of this parasite (Figure 2). Sequence analysis was conducted to discriminate between the classic or variant *P. ovale*. Basic local alignment search tool (BLAST) analysis revealed 95% similarity with *P. ovale* ssrRNA gene. The strain was classified as the classic type, *P. ovale curtisi*, based on its nucleotide polymorphisms [5]. The Genetics and Immunology Laboratory, Malaria Branch at Centers for Disease Control (Atlanta, US) confirmed these findings.

For malaria treatment he was prescribed 1,500 mg of chloroquine base given over 3 days and 15 mg of primaquine base over 14 days, following national guidelines for the treatment of *P. vivax* infections, as the results of the molecular analyses were not available at the time of treatment initiation. The patient was discharged after his clinical condition and hematological results improved. Follow up specimens were taken at day 1, one and two months after treatment completion, confirming parasite clearance. Also, all family members in the household and one neighbor provided blood samples, with negative results for *Plasmodium* spp. As a control measure, the household was sprayed with deltamethrin.

Here we present the first imported *Plasmodium ovale* case in Guatemala. Based on the patient’s travel history, he could have acquired the infection in any of the African countries he stayed in, as *P. ovale* is present in all of them [3,18]. Lack of strict compliance with both chemoprophylaxis and personal protective measures has been cited as a cause of imported malaria in deployed military personnel [19]. The patient’s late onset of symptoms corresponds to previous observations that anti-malarial prophylaxis delays, but does not prevent, the primary attack [3].

This report highlights the need to consider *P. ovale* as a cause of imported malaria and to include its detection, as well as *P. malariae,* in imported malaria screening programs. This patient presented particular diagnostic challenges that resulted in a failure of early detection and diagnosis of *P. ovale* infection. Distinguishing *P. ovale* from *P. vivax* by microscopy, already difficult due to the species’ morphologic similarity [20], was further confounded in this case by *P. ovale*’s non-indigenous status. Since this event, all returning military contingents are being tested with a pan-*Plasmodium* real time PCR assay. In addition to malaria screening programs, clinical diagnoses should not automatically exclude consideration of non-endemic species of malaria. Regular training of microscopists for the accurate identification of all malaria parasites, including non-indigenous species, should be mandatory [21].

In addition to mitigating the diagnostic challenges of imported malaria, monitoring imported cases into an already malaria-endemic area is important because the presence of viable vectors could lead to secondary autochthonous cases and the spread of drug-resistant strains, thus hampering malaria control and elimination efforts in regions, such as Central America that remains one of the few areas of the world where no antimalarial drug resistance has been reported [22]. An observed change in the number of imported cases or species of malaria could help re-direct prevention efforts both internationally and within Guatemala. Moreover, the combined use of molecular methods and microscopy will have the added benefit of possibly increasing detection of asymptomatic infections [23], allowing us to determine the true burden of infection and potential interactions among *Plasmodium* species [4].

Considering the long latency of these infections, further strategies should be considered to improve prevention. For instance, the use of presumptive anti-relapse therapy could be an effective approach to prevent relapses of *P. vivax* or *P. ovale* infections in long-term Army deployments which are significantly exposed to these parasites [24].

## Conclusions

Imported malaria cases could lead to the introduction of drug resistant strains in Central America, as well as atypical malaria species, especially in areas where competent hosts could harbor these parasites and transmit them [3]. An integrated and timely malaria surveillance system is warranted in order to rapidly detect and control the emergence of these imported infections in the country. In the era of malaria elimination, improved guidelines for the diagnosis, treatment and control of imported malaria cases are crucial in Mesoamerica to mitigate the risk posed by such novel cases of imported infections.

## Consent

Written informed consent was obtained from the patient for publication of this Case Report. A copy of the written consent is available for review by the Editor-in-Chief of this journal.
